# Reconfiguration, Contestation, and Decline: Conceptualizing Mature Large Technical Systems

**DOI:** 10.1177/0162243918768074

**Published:** 2018-05-01

**Authors:** Benjamin K. Sovacool, Katherine Lovell, Marie Blanche Ting

**Affiliations:** 1 Department of Business Development and Technology, Aarhus University, Herning, Denmark; 2 Science Policy Research Unit (SPRU), School of Business, Management, and Economics, University of Sussex, Sussex, United Kingdom

**Keywords:** sociotechnical networks, history of technology, large-scale infrastructure, megaprojects

## Abstract

Large technical systems (LTS) are integral to modern lifestyles but arduous to analyze. In this paper, we advance a conceptualization of LTS using the notion of mature “phases,” drawing from insights into innovation studies, science and technology studies, political science, the sociology of infrastructure, history of technology, and governance. We begin by defining LTS as a unit of analysis and explaining its conceptual utility and novelty, situating it among other prominent sociotechnical theories. Next, we argue that after LTS have moved through the (overlapping) phases proposed by Thomas Hughes of invention, expansion, growth, momentum, and style, mature LTS undergo the additional (overlapping) phases of reconfiguration, contestation (subject to pressures such as drift and crisis), and eventually stagnation and decline. We illustrate these analytical phases with historical case studies and the conceptual literature, and close by suggesting future research to refine and develop the LTS framework, particularly related to more refined typologies, temporal dimensions, and a broadening of system users. We aim to contribute to theoretical debates about the coevolution of LTS as well as empirical discussions about system-related use, sociotechnical change, and policy-making.

From birthing babies to managing old age, most of us remain intricately connected to large-scale, capital-intensive infrastructures (Misa 2003, 4) that are sometimes referred to as large technical systems (LTS). LTS are “machineries and freestanding structures performing, more or less reliably and predictably, complex standardized operations by virtue of being integrated with other social processes, governed and legitimated by formal, knowledge-intensive, impersonal rationalities” (Joerges 1988, 24).

LTS have become central to the modern human experience, yet they puzzle researchers and “confound engineers, social scientists, historians, economists, policy planners, and political leaders” (LaPorte 1991a, 1-2). Indeed, over time, LTS as a unit of analysis and the systems theories that informed their study have become less prevalent in the fields of science and technology studies (STS) and the history of technology. A “first wave” or “new direction” of LTS scholarship from Hughes (1983, 1986, 1987), La Porte (1991b), Gökalp (1992), Summerton (1994a), and Coutard (1999) offers much insight but is now decades old. Other recent work (Dafoe 2015; Schubert, Sydow, and Windeler 2013; Van der Vleuten 2004, 2009; Geels 2007) has nibbled on LTS themes but has not advanced an overarching conceptual framework or modified the “original” phases offered by Hughes or Gökalp. Still other recent work by Turnheim and Geels (2012) or Schot and Kanger (2018) emphasizes “phase models” to sustainability transitions, and Kanger and Schot (2016) as well as Schot and Kanger (2018) articulate “transition dynamics” according to a “start-up phase,” an “acceleration phase,” and a “stabilization phase” but do not situate these themes within the context of LTS.

Such lacunae lead us to ask: is the concept of LTS still useful for understanding the dimensions of technology or sociotechnical change? Do mostly sequential, fairly deterministic, phased conceptualizations of systems stand up to scrutiny? How do mature LTS evolve or decline? What happens after the establishment of momentum and style? What mechanisms or dynamics facilitate the further evolution or dissolution of LTS?

Our aim in answering these questions is to provoke more reflective thinking on LTS—exploring whether it still presents a useful conceptualization, how it may be modified to include recent intellectual developments, and how it can be further improved. We argue that mature LTS can move through the additional phases of reconfiguration, contestation (subject to pressures such as drift and crisis), and eventual stagnation and decline. To support our development of these analytical phases, we draw extensively from historical case studies as well as the conceptual literature. We aim to contribute to theoretical debates about the evolution of LTS as well as empirical discussions about system related use, governance, and policy-making. We also explicate hopeful new research directions for those seeking to further engage with the LTS heuristic.

## LTS: From Definitions to Conceptual Utility and Novelty

Although readers of this journal may be familiar with some of these themes, here we outline the LTS concept, explain its use, and discuss its operationalization through five key attributes before we argue why it remains conceptually useful. We then seek to expand and elaborate components of LTS theory.

### Defining an LTS: Society, Scale, Coordination, Variation, and Obduracy

Despite repeated attempts, the literature does not offer a concise or universal definition of LTS (Hughes 1983, 1987, 1992). Van der Vleuten (2004) argues that there is no agreement concerning what constitutes LTS, with some talking about “society-wide infrastructures,” others “nodes and junctions,” and still others “material superstructures” or “loosely coupled systems.” In later work, Van der Vleuten (2006, 281) even notes that he “cannot offer the reader a strict definition of large technical systems here, simply because there is no consensus.” Here, we argue that LTS have four attributes: they are sociotechnical, large, coordinated, and varied. Mature systems, in addition, are obdurate.

The *social and technical* elements of LTS are diverse, including technological infrastructure as well as political, regulatory, financial, educational, and other social dimensions. To offer two examples: LTS for electricity will involve not only coal mines, power plants, electric transmission and distribution lines, transformers, and pylons but also financing institutions, regulatory bodies, technical universities, electrical engineers, and residential and commercial users. LTS for transport will involve not only cars and roads but also traffic signals, fuel stations and refineries, the maintenance industry, registration offices, insurance companies, drivers, passengers, and even police and legal networks. To be sure, this conceptualization complements a longer line of historical thinkers all positing that many activities possess a “sociotechnicality” highlighting integration of physical hardware with the human environment or software needed to utilize it (Trist and Bamforth 1951; Emery and Trist 1960; White 1962; Trist 1981; Hughes 1983).

In *scale*—LTS are spatially expansive and capital intensive. They require billions or even trillions of dollars of investment and occupy substantial areas of the physical environment. LTS therefore involve and change the social lives of large numbers of people; some, like early railroads, even restructured social life and conceptions of space-time, something Gökalp (1988) termed a *secteur-reseau* (network sector) to denote its macrolevel influence. Not all LTS may be deeply penetrating, but the idea is that LTS are big and dominating in their reach.

LTS are also *coordinated* “goal-seeking systems” (Hughes 1983, 80), composed of related parts, nodes, or components that are structured or connected, often centrally, to achieve some sort of task. Van der Vleuten (2006) adds that LTS structure various social, educational, scientific, and even religious subsystems, making them centrally coordinated and hierarchically organized. This arrangement creates significant “junctions” where systems can overlap and interact, leading to collaboration (such as when maritime navigation or air transport is connected to land transport via harbors and airports) to cooperation (when railway systems are interlaced with electricity supply systems). But LTS retain a goal-seeking or functional nature even as it may compete with other LTS.

LTS have *varied* technical architectures that organize interactions among diverse actors and technologies to accomplish a variety of purposes. Some LTS distribute water or electricity, others accumulate waste or sewage, and still others organize communication or transportation. This variety leads to variations in form and function, governance, institutional management, and style (or cultural variation), both within and across systems.

The fifth and final attribute of LTS, which arises in their maturity, is *obduracy*, or resistance to change (Hommels 2008). Hughes (1983, 15) described this characteristic as “momentum,” or as a mass of “machines, devices, structures” and “business concerns, government agencies, professional societies, educational institutions and other organizations” that “have a perceptible rate of growth or velocity.” Joerges (1988) termed it “dynamic inertia,” others refer to it as “path dependence” or “lock-in” (Kirsch 2000; Unruh 2000). These terms all describe how LTS continue along a given path, reflecting the actions of numerous stakeholders such as educational and regulatory institutions, engineering and equipment suppliers, and the work and culture of people working within an industry. Managers of LTS contribute to inertia by remaining in control and resisting new and disruptive technologies (Rip 1995). Since momentum and inertia tend to direct systems along established lines of development, Hughes (1983) points out that it takes massive contingencies, such as war, to disrupt that momentum. Others, such as Hirsh (1999), note that social and regulatory movements, tied with technological change, can also alter momentum significantly.

### Conceptual Utility: Structure, Agency, and Meaning in LTS Evolution

What makes LTS a valuable concept, given other advances in sociotechnical conceptualizing? The central focus of LTS theory is the system itself, and so applications of LTS thinking tend to emphasize structure, or how LTS exert a “soft determinism” that constrains human agency and influences meaning or discourse (Sovacool and Hess 2017). Hughes (1989) even argued that modern American society was built of intertwined LTS that laid a material foundation for an entire civilization—the LTS essentially influenced social change. LTS reflect “deep structures” in society that can surpass natural geography or politics as key drivers of societal change (Van der Vleuten 2006; Van der Vleuten et al. 2007). Although they may overemphasize successful top-down alignment of systems and underemphasize conflict and failure, LTS theories convey the notion that technology can have deterministic effects. For example, the operation of LTS involving fossil-fueled electricity or transport cause greenhouse gas emissions and life-endangering pollution, and the design of Dutch drainage canal networks shaped patterns of human habitation and agricultural productivity for centuries to come. Soft determinism does not necessarily mean LTS will forcefully dictate social change, only that they can act as a “force field” for such changes.

LTS theory assumes that such systems undergo mostly sequential phases. Although he suggests they overlap and are not always linear, Hughes (1983, 1987) proposed that LTS progress approximately through phases from invention and technology transfer to growth before becoming directed by established momentum and style. *Invention and development* occur when an inventor or entrepreneur thinks that a novel product, good, or service has significantly improved characteristics or uses and so develops it, and then makes the necessary connections with engineers and financers. For those inventions that develop beyond the first phase, what Hughes called *technology transfer* occurs—the successful diffusion or exportation of a technology across space or between societies. The phase of *growth* refers to extending the scale, scope, or speed of the system, increasing capacity use and other performance measures; it is more difficult to characterize, as it relates to the deepening and specialization of systems. The phase or mechanism of *momentum* building includes notions of lock in and incumbency that come to foreclose choices and trajectories so that system development is based upon past conditions and decisions. The phase of *style* emphasizes that LTS can exhibit a distinct style formed by conditions beyond or external to the system itself (including geography, economic structure, cultural values, legislation, and contingent historical factors).

However, while LTS theory concerns itself mostly with macrotechnical or deep structure and phases of development, there is also space for agency and meaning. LTS involve individual or organizational/collective system builders who, at times, are users of the system as well as designers. The work and art of system building reveals the human mechanisms of LTS, and gives rise to useful concepts such as reverse salient (component(s) out of equilibrium impeding system performance) or load factor (extent of system capacity used in delivering a product or process). Van der Vleuten et al. (2007, 4) note that the notion of system building “humanizes” infrastructure studies and history, and replaces the traditional “heroic” narrative of brilliant inventors with a more complex narrative of dedicated teams of system builders. As they write, “The system builder concept…invites historians to follow key actors as they routinely cross disciplinary boundaries and engage in transdisciplinary problem solving while building sociotechnical systems.”

LTS theory also enables the analysis of meaning and discourse as they arise in system evolution. In their review of the wider LTS literature, Van der Vleuten et al. (2007) found that system builders often framed their infrastructure discursively or connected it to broader rhetorical or ideological agendas. This element opens up LTS inquiry not only around patterns of structure and agency but communication strategies, promises and expectations, and the negotiated and contested rhetorical politics of system development.

These features distinguish LTS theory from other sociotechnical concepts. As Table 1 shows, the multilevel perspective (MLP) analyzes sociotechnical transitions but emphasizes regimes, dominant routines, and alternative spaces or niches. While it discusses global or landscape trends, it is far less deterministic—and linear—than LTS thinking. The MLP also has a different actor concept: system change is not happening because of the work of system builders as such but because of the emergence of niches and exploitation by networks of actors. Actor Network Theory invokes concepts such as “network assemblages” and “sociotechnical imbroglios” but focuses more on agency or politics, especially at the microlevel. The Social Construction of Technology (SCOT) emphasizes closure, frames, and the meaning groups of stakeholders give to technology. SCOT focuses on the evolution of particular technologies rather than the evolution of a system, and it looks less at how that system shapes and constrains agency and evolves over time. Technological Innovation Systems (TIS) do assess complexity and variation in large systems but prioritize the functional aspects of innovation. TIS theories tend not to discuss sociotechnical change in the “big picture” perspective of all other approaches and instead link to the shorter time management of innovation in particular sectors (five to fifteen years’ time horizon).

**Table 1.** Five Sociotechnical Conceptual Approaches.

Theory/Concept	Discipline(s)	Emphasis	Key Concepts	Key Authors
Multilevel perspective (MLP)	Evolutionary economics, sociology, innovation studies, and STS	Transitions: sociotechnical system change	Niches, regimes, and landscapes	Arie Rip, Frank Geels, Johan Schot, and René Kemp
Actor network theory (ANT)	Sociology, STS	Agency: how actors (human and nonhuman) build and become entangled in actor networks	Network assemblages, translation, enrollment, entanglements, and politics	Bruno Latour, Michel Callon, John Law, and Steve Woolgar
Social construction of technology (SCOT)	STS, history of technology	Meaning: how different groups of social actors interpret technical artifacts, systems or services	Interpretive flexibility, relevant social groups, technological frame, closure, and heterogeneous engineering	Wiebe Bijker, Donald MacKenzie, and Trevor Pinch
Technological innovation systems (TIS)	Innovation studies	Innovation: the interconnected functions that promote or constrain technical development	Knowledge development and diffusion, entrepreneurial experimentation, broader political and social influence, market formation, legitimation, resources mobilization, and positive externalities	Staffan Jacobsson, Anna Bergek, and Marko Hekkert
Large technical systems (LTS)	History of technology	Systems: large-scale, capital-intensive sociomaterial systems and subsystems	System builders, momentum, reverse salient, load factor, and vertical and horizontal coupling	Thomas Hughes, Jane Summerton, Oliver Coutard, Todd La Porte, Iskender Gökalp, and Erik van der Veuten

*Source:* Authors.

*Note*: STS = science and technology studies.

### Novelty: Theorizing Mature LTS

The balance of the paper explores the evolution of LTS beyond the five phases articulated by Hughes, and how such an extension offers benefits for understanding modern technological enterprises. It suggests that LTS can may be reconfigured as system builders adapt to dynamic challenges to retain control or extend quality of service, system reach, or volume. Contestation occurs when control or function is challenged. Decline occurs when a system deteriorates. We explore each of these phases using historical cases to develop concepts for the study of mature LTS. Our notion of phases recognizes that technology is not freely transferrable from one situation to another but instead mediated, acquired, appropriated, and modified. Table 2 provides an overview of our framework.

**Table 2.** Phases, Mechanisms, and Empirical Cases for Reconfiguration, Contestation, and Decline.

Phase/Description	Mechanism(s)	Case(s)
*Reconfiguration:* system adapts to challenges; control over system is mostly stable	Interconnection and crosslinking	Railways, electricity grids, and telecommunications networks
	Selectivity	Electricity grids, telecommunications networks, and gas pipelines
	Repositioning	Sewer systems, ocean freight and marine transport, land transport, industrial manufacturing, and natural gas systems
*Contestation:* system is in limbo; control over system is challenged	Drift	South African electricity, shale gas in Eastern Europe, and telecommunications in the United States and United Kingdom
	Crisis	American flood control, British railways
*Stagnation and decline:* system growth declines or erodes; quality of service or volume deteriorates; control over system is lost	Substitution and transformation	French railways, electric streetcars (trolleys) in the United States, and coal in the United Kingdom

*Source:* Authors.

*Note:* Particular mechanisms often appear across multiple phases. However, phases reflect where certain mechanisms dominate.

## Reconfiguration: Linking, Selection, and Repositioning

Although mature LTS can create powerful feedback mechanisms that resist change, these are neither inevitable nor fully deterministic. Over time, LTS evolve and at times struggle as they face internal and external pressures. Systems may be reconfigured through territorial interconnection and cross-linking, unselecting undesirable users, or realigning and dealigning.

### Interconnection and Cross-linking

The three types of LTS reconfiguration discussed by Summerton (1994a, 1994b, 1999) offer a useful starting point to understand changes connected to space, function, and organization. Geographic reconfiguration can occur with territorial expansion and interconnection of similar systems across political boundaries. Summerton calls this the territorial coupling of autonomous systems, or a meeting of the systems, whereby independent regional or national systems are physically connected and standardized. This type is primarily about transforming systems into national and international ones, reconfiguring their geographic size in ways that may include growth or shrinkage. Functional reconfiguration occurs during attempts at full-system integration or organizational mergers that combine complementary parts of different systems into a new whole such as transportation systems altered by linkages with communication and energy systems (e.g., telegraphs, electricity, and railways). A final type of reconfiguration occurs when monopoly systems reorganize by blurring their institutional borders rather than by altering spatial or function boundaries. Regardless of whether reconfiguration is spatial, functional, or organizational, Summerton argues that LTS interlink in ways that allow actors to integrate and coordinate complementary resources and skills, without sacrificing autonomy or control, to harmonize interests and/or maximize profits.

Exemplary cases of reconfiguration involve telecommunications following the fall of the Berlin Wall in Germany (Robischon 1994), the co-development of electricity and railway (Mulder and Kaijser 2014), and the liberalization of the European electricity sector (Markard and Truffer 2006). In other cases, LTS can be suddenly delinked. An example here is the Cold War, which Misa and Schot (2005) interpret as “delinking” transport, energy, and communication systems in the middle of geographic Europe. Such boundary changes represent a sudden shift in the *center of gravity* of LTS, redirecting their trajectory. They bring with them changes in actors, knowledge bases, interests in the system, and the characterization of the environment at the system boundary (i.e., its position within the society it serves).

### Selectivity

The cross-linking and interconnection of LTS into larger scales that serve bigger geographical territories is not only about space or scale—it can empower system operators or owners to more easily shape the markets they serve. Such expansion can enable more strategic activity designed to increase profits for operators and financiers. Once networks reach a certain size, it becomes possible to increase profitability by concentrating only on the most lucrative customers and markets, something Guy, Graham, and Marvin (1996) and Guy, Graham, and Marvin (1997) term “cherry-picking”; or to “dump” unprofitable market segments.

In the United Kingdom, the expansion of electricity, natural gas, and telecommunications networks enabled utilities to focus on large commercial users and more coverage in market “hot spots.” For instance, Guy, Graham, and Marvin (1999) note that interconnection made possible the process of “social dumping,” empowering service providers to ease out of unprofitable areas, letting go of marginal customers who are no longer essential to profits such as those in rural areas or impoverished pockets of the inner cities. Graham and Marvin (1994) similarly documented social dumping practices such as line rental and service charges, high deposits or prepayment systems, and disconnection of the poorest customers among UK utilities. Graham (1997) identified social dumping practices such as socially regressive tariff rebalancing, “self-disconnecting” prepayment meters, and “smart” meters. Essentially, making LTS larger can embolden operators to withdraw from zones of unprofitable activity. Moreover, when new consumers become dependent on the newly expanded system, operators and owners can begin to push them to modify their patterns of consumption and habits in order to increase use and/or profitability.

Patterns of selectivity are not limited only to Europe. Kline (2002) and Cannon (2000) noted a tendency for companies in the United States erecting electricity networks into rural areas in the 1930s and 1940s to “skim the cream” by rapidly moving to supply densely populated areas or wealthy farmers, and avoiding more sparsely populated areas or poorer communities. British Gas and BT in the United Kingdom have also attempted to selectively pick international segments of customers via strategic arrangements with service companies in global markets (Graham and Marvin 1994).

### Repositioning

Multiple causal drivers can force LTS into reconfiguration (Geels 2007), drivers that we place under the umbrella term of “repositioning.” Beyond changes responding to underlying problems within the system that acquire the urgent attention of managers, there are challenges emanating from outside the system. Examples include concerns about safety or environmental externalities, changing competitive environments, political contingencies, or shifts in cultural values and consumer behavior. At some point, these can create pressure and shock LTS into various transition pathways (Geels and Schot 2007; Schot and Geels 2008). Geels and Schot (2007) present a typology, summarized in Table 3, based upon the extent and speed of environmental shifts and upon the system’s readiness to adjust.

**Table 3.** Features of Repositioning Described as Sociotechnical Transition Pathways.

Pathway	Main Actor(s)	Types of Interaction
Transformation	Regime actors, outside groups, and social movements	Outsiders voice criticism, and incumbent actors adjust regime rules
Technological substitution	Incumbent firms, new firms	Newcomers develop novelties that compete with regime technologies
Reconfiguration	Regime actors, suppliers	Regime actors adopt component—innovations, developed by new suppliers; competition occurs between old and new suppliers
Dealignment and realignment	New niche actors	Changes in deep structure create strong pressures that challenge faith and legitimacy, followed by the emergence of multiple novelties and competition; eventually one wins, leading to restabilization

*Source:* Modified from Geels and Schot (2007).

Geels and Schot (2007) offer numerous examples illustrating repositioning through these pathways. The transition in the Netherlands from cesspools to sewer systems depended on the emergence of new social norms about cleanliness, and system development responded directly by incorporating rules about disease and waste, altering the role of public authority, and changing waste disposal practices. The transition from sailing ships to steam ships in Britain illustrates a pathway of technological substitution, keeping the main incumbents of oceanic freight in control. The transition from traditional factories to mass production typifies a reconfiguration pathway, as it relied on the replacement of manual or animal labor with automated machinery, assembly lines, and mechanization. The transition from horses to automobiles shows the development path of an existing system undermined, and a considerable period of uncertainty before the system builders, knowledge bases, and a system development trajectory around automobiles became established.

Geels and Schot (2007) frame their discussion in terms of sociotechnical transitions; as a result, they restrict discussion of the means of establishing new rules for system development to the creation and expansion of a protected space or niche. When applied to LTS, we can acknowledge other ways for those rules to be established. For example, new development practices can arrive through the entry of new system builders with different interests and practices, perhaps applied or developed coherently in another setting. Alternatively, new opportunities for LTS, such as new sources of system inputs, can provoke realignment processes, and they can be responded to without protected spaces or niches, a case in point being Kaijser’s (1999) study of changes to the gas industry in the Netherlands.

Such LTS pathways can provoke different social responses. La Porte (1991a) has noted four distinct and, at times, contradictory responses. Some societies respond by giving operators complete, independent control. Some develop governmental subsidies and legal protections that sponsor technological growth. Others develop analytical capacity to forecast the effects of systems in an attempt to design away undesirable effects. Still others create regulations to moderate the behavior of system operators, and some enforce punitive economic and legal regulations after damage of systems become evident. These social responses can further alter LTS development and add to the complexity and variety of the possible paths of repositioning.

## Contestation: Drift and Crisis

LTS undergoing reconfiguration do not always result in consensus—they can invoke compromise or at other times outright conflict and contestation. The stakeholder frames attached to LTS become fragmented to the point where they clash; where a lack of “cognitive consensus” (Schot 1992, 20) about function or meaning arises. This relationship between contestation and reconfiguration is iterative and dynamic: sometimes, contestation can shift systems closer to decline (a negative impact on the system); in other situations, it can shift them back toward reconfiguration (a positive impact).

Interestingly, this dynamic relationship has been captured by emerging work in the sociology of infrastructure. Bolton and Foxon (2015) write that many LTS go through an “infrastructural lifecycle,” as they call it, which is cyclical—it involves a constant didactic process of decay and renewal. This notion of cyclical development is also encapsulated by Gökalp (1992) who writes that sometimes contestation can lead to decline, but in other situations, it can convince system managers to adopt the characteristics of new innovation “threats” so that they are contained and utilized to affirm the revitalization of the older system. Sovacool et al. (2017) similarly noted how incumbents in the automotive industry sought to “contain” emerging innovations in electric mobility related to vehicle range and charging.

In the remainder of this section, we talk about two particular mechanisms that can provoke contestation: drift and crisis. To recap: contestation refers to the challenge of control over the system, where entities dispute, contest, compete, and contend some aspect of LTS functioning, placing them in stasis or jeopardy. Decline refers to when LTS exhibit stagnation or declining growth, often because control over a system is lost. Decline can happen through various means without contestation, and not all contestation can lead to decline.

### Drift

The term “drift” initially stems from institutional theorists describing efforts by incumbents holding on to the status quo despite major shifts in contextual relevance (Hacker, Pierson, and Thelen 2013; Streeck and Thelen 2005). Hacker, Pierson, and Thelen (2013, 28) elaborate that with drift, change occurs not as an “electoral spectacle” or “big legislative battle” but instead away from public oversight and through quieter, less-prominent channels. Drift suggests that mature LTS are constantly maintaining their suitability to their present context, but this often entails clashes with groups of actors with divergent interests. Drift occurs when relevant social groups wage a battle or contest for control over the system, or when system operators or developers take an overly cautious, even counterproductive approach to steering LTS, what Lorenz (1994) called “collective conservatism.” Three historical examples of drift relate to centralized electricity networks in South Africa, natural gas networks in Eastern Europe, and telecommunications in the United Kingdom and United States.

South Africa’s electricity supply system reveals how an entrenched incumbent, Eskom, has come to find itself in drift, faced with the growing influence of independent power producers. Eskom is the state-owned, vertically integrated monopoly with regulatory and technical control over almost all of South Africa’s electricity system, including power plants, transmission and distribution networks, tariffs, and licenses (Ting 2018). Eskom has ambitious plans to further enlarge its system via investments in regional power pools and pan-African electricity supply centered on large-scale hydroelectric dams (Green, Sovacool, and Hancock 2015). Starting in 2008, Eskom’s control has been challenged by decentralized, independent power providers, often relying on renewable sources of supply such as wind turbines and solar photovoltaics. Under the 2011 Renewable Energy Independent Power Producers Procurement Programme (RE IPPPP), more than 5,000 MW of capacity across seventy-seven independent power projects has been procured. This has resulted in renewable energy bid prices for solar photovoltaic panels and onshore wind turbines dropping by more than 50 percent in three years (Ting 2018). Eskom has attempted to reassert control over rules concerning interconnection and integrated resource planning by delaying and resisting the conclusion of power purchase agreements emerging from RE IPPPP. Contestation between Eskom and renewable energy developers is placing the sector in limbo. The dominant centralized grid dynamic of the system is losing significance, and distributed renewables, especially rooftop solar panels, are gaining influence. At present, it is not certain whether drift will result in the reconfiguration of the incumbent or the beginning of stagnation.

Similarly, drift describes the fragmentation of consensus related to the system of natural gas networks—involving horizontal drilling sites, production facilities, transmission and distribution pipelines, and end-use facilities such as industrial enterprises (chemicals, steel, refining, compression, and liquefaction), power generation, and even direct household use (gas for cooking)—in Bulgaria, Poland, and Romania. These countries face the choice of embracing shale gas into their LTS as a domestic source of energy against the backdrop of Russia serving as the dominant gas supplier (Goldthau and Sovacool 2016). Powerful stakeholders in industry and government have been backing a shale gas sector based on the reindustrialization of the economy that it could provide, along with geopolitical stability by displacing Russian imports. Conversely, equally influential stakeholders with competing industries (such as those for renewable energy) and civil society groups have countered that shale gas threatens water quality and availability, risks chemical pollution, and will accelerate species loss and the destruction of habitats. They also note that shale gas production merely transfers wealth and revenue out of domestic economies to foreign actors. It is yet unclear whether this contestation will lead to the decline or reconfiguration of shale gas supply within the energy system.

Telecommunications in the United Kingdom and United States (Davies 1996) is a final example of drift. There, a contest for control manifested during the 1970s and 1980s as an “electronic alliance” of large corporate telecommunications users and electronic data processing companies pushed for deregulation and restructuring. These efforts ran up against a “postal–industrial complex” composed of a coalition of telephone companies, national equipment suppliers, and trade unions. Such conflict placed the system in relative drift to the point where new business models and technologies, namely, cellular telephone and digital providers of Internet services, were able to threaten the hierarchical and centralized network of landline telephones. This contest led to reconfiguration: the evolution of a new hybrid system whereby overlapping local access networks controlled by different operators act as the spokes feeding traffic into high-capacity hubs.

### Crisis

Crisis occurs when LTS are placed into contestation rapidly over a major accident or external event.

British rail networks offer an illustrative case example. Shortly after railway privatization and restructuring (1993-1997), a series of railway accidents with fatalities occurred. Investigations into these incidents found problems with infrastructure maintenance and control of contractors (Cullen 2000, 3-4). The Hatfield accident (October 17, 2000) is considered a watershed moment for the sector. Caused by a broken rail, the accident “threw the industry into something resembling organized chaos” (Gourvish 2008, 59). The accident prompted the introduction of speed restrictions across the rail network: eighty-one sites had emergency speed restrictions added on the day of the accident; a week later, 1,850 sites with cracks had been found and speed restrictions were introduced at 272 sites. By the end of November, cracked rail sites were up to 3,400 and 850 had speed restrictions (Gourvish 2008, 68). The problems identified by the accident resulted in significant institutional and financial restructuring of the entire railway system.

In other situations, natural disasters can lead to crises that prompt reconfiguration. Consider the system involving flood protection or “flood hazard mitigation” surrounding New Orleans, LA. When Hurricane Katrina struck New Orleans in August 2005, the storm breached the floodwall of the Lower Ninth Ward, causing multiple other levees and flood barriers to fail, ultimately covering more than 80 percent of the city in water as high as ten meters. The federal government allocated billions of dollars to the Army Corps of Engineers to fix, upgrade, and rehabilitate about 220 miles of levees and floodwalls, floodgates, pump stations, and canals, spending a budget of $14 billion (Sovacool and Linnér 2015). This led to a radical reconfiguration of flood-level protection. To expedite repairs, environmental and air pollution standards were curtailed so that hazardous wastes were not properly stored and bans on open burning lifted (Sovacool and Linnér 2015). The rebuilding of canals and roads further eroded environmental buffers (such as wetlands) critical to future storm surge mitigation (Sovacool and Linnér 2015). Repairs were never fully implemented by the US Army Corps of Engineers in levees closest to many rural and minority areas, meaning people moved back into unsafe areas (Sovacool and Linnér 2015). Reassurances offered by public officials about the safety of the reconfigured system resulted in a pattern of rebuilding, which integrated living patterns within levees and protective infrastructure such as canals, polders, and dikes, in actuality undermining their ability to withstand the impacts of severe storms (Kates et al. 2006). Thus, the system was reconfigured in ways that increased the inequality of protection. By diffusing responsibility for flood protection (Wetmore 2007), the system masked the way it redistributed risk among vulnerable people. A positive outcome is that it did provoke “new imaginings” about managerial visions of flood control that created a sense of disturbance, crisis, and political damage (Hilgartner 2007).

## Stagnation and Decline

The final phase is that of decline: when LTS see growth slow and stagnate, eventually coming to be displaced or substituted by other competing systems. Decline can be absolute, compared to previous levels of system plateaus, especially when they suffer from technological stasis (Hirsh 1989), or relative to other competing LTS (Lorenz 1994). Although decline can be a matter of perspective—one system’s decline may be another’s fruitful reconfiguration—here, we assess decline by using measures such as an actual loss of service (quality or volume) or a shrinkage of geographic scale. Three examples are offered: French railways, electric light rail (trams) in the United States, and the coal supply system in the United Kingdom.

In France, although the rail network hasn’t disappeared completely, the closure of certain rail lines proceeded in parallel with the accelerated development of motorways, trends coupled with a shift in preferences for personalized, motorized transport in cars and a significant decline in both numbers of users/volume and the geographic reach of the rail network. Highways for cars accounted for only about 10 percent of transport of goods based on land in the 1920s, but this rose dramatically to above 50 percent by the 1970s; over the same time, the percentage of goods carried by rail dropped from above 70 percent to below 40 percent. Although some hypothesized a resurgence of French rail in the early 1990s due to the development of high-speed lines such as the TGV, Gökalp (1992) countered that in fact improved performance of cars more than offset such a push, with cars becoming increasingly computerized both in terms of their energy sources (computer control of combustion) and in terms of their network (computer-aided signaling and traffic control). French rail therefore became largely displaced by French cars.

In the United Kingdom, the coal system involving coalmines, transport and logistics networks, storage facilities, pollution control devices, coal-fired power plants, and industrial factories has seen an accelerated decline from the 1930s to the 2010s. Already in gradual decline over the previous decades, the negative pressures against coal accelerated in the 1950s as the coal supply system entered a period of crisis connected to shortages in supply and rationing, as well as visible environmental calamities such as the “big smog” of 1952 that resulted in thousands of excess winter deaths (Fouquet 2012). Public perception shifted to frame coal as old-fashioned, dirty, and outdated, and government responses such as the passage of the Clean Air Acts further intensified the rate of mine closers and channeled investments to fewer enterprises. National energy policy pivoted to a “four-fuel economy” that saw a greatly reduced role for coal and an encouragement of users to switch to gas for heating (Turnheim and Geels 2012). The creation of “smokeless zones,” and the prospect of cheap imported oil from the Middle East, saw an even quickened conversion to other fuels in the 1970s. Intense pressure against coal with the election of a newly Conservative Government led by Margaret Thatcher in 1979, who saw coal (in particular coal miners) as an example of a monopoly acting against the efficacy of market forces, continued the decline. The privatization of the electricity supply industry in the 1990s further hastened the decline of coal, culminating in a so-called dash for gas that ended up displacing fifty million tons of coal production (Turnheim and Geels 2012). The industry suffered “extreme contraction” and employment fell to only 10,000 miners (Fouquet 2008); production of coal dropped precipitously from forty-four million tons in 1995 to only nine million tons in 2015, as nuclear power and natural gas further displaced coal for electricity supply.

**Figure 1.**
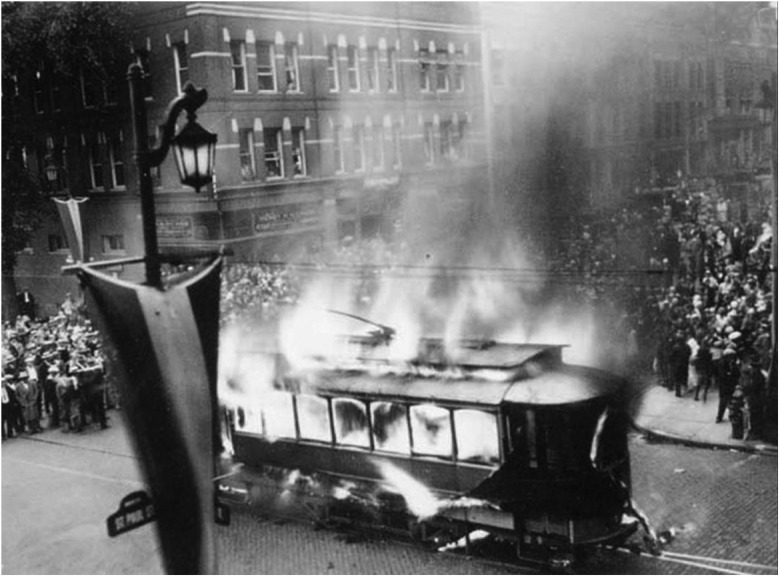
A “funeral pyre” for an Electric Street Trolley in Vermont, 1929.

A third example is the decline in service volume of electric street trolleys (trams) in the United States, a system involving light rail as well as electricity, transport, road, and carriage networks. Although public transportation did not affect most Americans until the arrival of the electric streetcar in 1888, streetcars developed rapidly after its introduction (Slater 1997). By World War I, there were few towns of more than 10,000 population without a streetcar system. Prior to 1920, streetcar use increased steadily, stimulated by rising incomes, lower real fares, and rapid urban population growth. These positive influences overcame the negative effect that increased automobile use had on streetcar ridership. However, threats to streetcar dominance began to emerge around 1914-1916, when gasoline powered jitneys—unlicensed, informal taxicabs, very similar to Uber today—made serious inroads into streetcar ridership until legal maneuvering by the streetcar companies put most of the jitneys out of business. The result however was a rapid shift to vehicles powered by liquid fuel rather than electricity, and the impact on the streetcar companies was severe. Some companies lost as much as 50 percent of their ridership. A more lasting decline was precipitated by the further refinement of adoption of commercial buses as well as private cars. The modern motor bus saw fairly systematic innovations to its chassis and engines, which resulted in improvements in speed, handling, and comfort. Buses attracted new ridership because they were much faster and more comfortable than streetcars, particularly after the introduction of the heavy-duty pneumatic “balloon” tires during the early 1920s. Buses were also safer since they could pull in to the curb to discharge passengers, whereas streetcars let passengers off in the center of the street. In 1914, streetcars provided 100 percent of US cities’ public transportation, but by 1937, only 4 percent of US cities with public transportation were served by only streetcars; 50 percent of cities were served only by buses. A final cause of streetcar decline was that automobile ownership grew from 8.1 million in 1920 to 23.1 million in 1929 (Slater 1997). Occasionally, the demise of the streetcar was celebrated—Figure 1 shows the literal burning of the last streetcar in Burlington, Vermont, in August 1929, with people celebrating their “liberation” from the streetcar and the lure of the freedom and independence of the automobile (Vermont Historical Society 2004).

## Research Frontiers: Typologies, Temporalities, and Users of LTS

As the examples above illustrate, the evolution and progression of LTS can involve both multidimensional and interactive phases and mechanisms. In some situations, users can change their preferences away from a particular technology that invokes contestation or decline, such as a preference for cars leading to the decline of railways. In still others, systems reconfiguration occurs strategically and dynamically, interconnecting with other LTS, cherry-picking customers, or repositioning business models or services provided.

Clearly, LTS are diverse in their functions, subcomponents, services, users, institutions, discourses, contextual drivers, and struggles. Toward that end, we offer three directions for new research. This list is not exhaustive; our intent is to push and provoke more refined and reflective thinking about LTS methodology, theory, and empirical application.

### Dichotomies and Typologies of LTS

In his review of the literature, Van der Vleuten (2006) offers multiple ways one can classify LTS. One can demarcate systems by their technical, geographical, economic, and institutional properties, the result of which could be typologies or pathways of development patterns. For instance, electricity grids and railways have specific networks; maritime navigation, telecommunication, and air traffic use nature-based links to interconnect to human-built nodes; others such as postal systems use existing networks to link artificial nodes. Some LTS are grid based, such as electricity and rail, whereas others are loosely coupled, such as water control or aviation. LTS can even compete with each other for the “five Ps” of price, performance, political pressure, legal protections, and propaganda.

Other ways of categorizing LTS center on openness, capital and labor, or layering. Both Sovacool (2010) and Kraemer (2006) argue that systems can be characterized as “open” (more inclusive, democratic, flexible, and decentralized) or “closed” (exclusive and proprietary, authoritarian, rigid, and centralized). Some LTS are more capital- or labor-intensive than others, taking longer to build and being more obdurate to change, such as canals versus naval shipping networks, or flight paths for airplanes. Another distinction is between “first-order” and “second-order” LTS: second-order LTS are constructed by combining familiar first order LTS to create a new network or function. Braun and Joerges (1994) use the example of a transborder organ transplant system. This is a second-order system, as it relies on the blending of national medical systems (hospitals), telecommunications systems (satellites and mobile phones), and energy and transport systems (helicopters, planes, cars, etc.). The integration of European militaries into a holistic system of industrial mass warfare during World War I is another example of a second-order system requiring the integration of command structures, contractors, railways, and information systems such as telegraphs and telephones (Bucholz 1994). Other examples of second-order LTS include mass tourism, global stock exchanges, and shipping container systems, all erected “on top of” existing transport, communication, or energy systems (Van der Vleuten 2006).

This leads us to ask: which typologies of LTS offer the most explanatory power or rigor? How can we test, validate, or challenge such typologies with empirical data? Do new typologies arise as LTS enter the phases we elaborate above such as reconfiguration, contestation, and decline? Which typologies are most or least likely to induce infrastructure change? What implications does this have on accelerating or overcoming change?

### Temporalities of System Progression

The temporality of LTS is a second key area deserving of more analysis. We have begun to sketch some of the later phases of “mature” LTS in this article, but even so, historical LTS may differ fundamentally from contemporary LTS, which may differ further still from the LTS that humanity will come to adopt in the future. Most of the examples we provide above are from 1880 to today. Only flood protection in the United States, coal in the United Kingdom, and rail privatization in the United Kingdom involve changes to LTS after 1990. Mature systems may diverge from emerging systems in the same way an adult’s body differs from a child’s. Hughes (1983, 1987) also suggests that social scientists have given inadequate attention to temporality in their analysis of LTS in the past. In his examination of sociotechnical transitions across energy and transport systems, Sovacool (2016) proposes that while the previous historical drivers of transitions emphasized abundance and changes in supply, the future drivers may shift to scarcity and changes in demand preferences. Similarly, Kern and Rogge (2016) suggest that the pace and speed of future transitions across sociotechnical systems may be about actively altering the selection environment to accelerate change rather than historical patterns of crisis-driven or market-driven progression.

The temporality of LTS lead us to ask: are changes to LTS bound with particular innovation environments prevalent within certain periods of history (e.g., modernity)? How can LTS characterized by path dependence or momentum embodied in past values be changed in a current context? Are historical cases predictive of future LTS evolution or merely informative? Which policy mixes can dismantle or actively phase out undesirable mature systems so as to provoke their decline? Do sociotechnical systems evolved in the deeper past respond differently from those created today? Are different typologies needed for historical versus modern or future LTS?

### Users beyond System Builders

We classify LTS as a structure-centered theory that also has compelling relational elements incorporating agency and meaning. Agency is discussed, but often via the notion of system builders. Recent work, however, has begun to elaborate more refined ways that users may exert influence over sociotechnical change (Oudshoorn and Pinch 2003) and thus LTS progression. Kivimaa and Martiskainen (2017) and Martiskainen (2017), for example, identify “user intermediaries” (actors or users who influence other users or the selection environment), whereas Parag and Janda (2014) discuss the role of “middle-out actors” in sociotechnical change. Schot, Kanger, and Verbong (2016) argue that at least five types of users exist:

- User-producers create new technical and organizational solutions;
- User-intermediaries shape the needs and desires of users as well as products, infrastructures, and regulatory frameworks;
- User-citizens engage in politics of regime shift lobbying for a particular niche;
- User-legitimators shape the values and worldview of niche actors;
- User-consumers appropriate products and services and thus producing meaning and purpose, and testing new systems.

Within these categories, intermediary users come closer to “system builders” as they shape a mediation junction but are not limited as such. Battilana, Leca, and Boxenbaum (2009) add that some users may leverage resources to create new or transform existing institutions; Johnstone, Stirling, and Sovacool (2017) retort that other users may attempt to capture resources to further entrench patterns of incumbency. Hoogma and Schot (2001) differentiate “lead users” from others in terms of their competency, resourcefulness, and enthusiasm for innovation; Lindsay (2003) discusses “reflexive users” who imagine and define future users (and applications) in their own image. Users can even actively resist and oppose particular LTS such as telephones and rural electricity networks (Kline 2003) or smart meters for gas and electricity (Kahma and Matschoss 2017; Sovacool et al. 2017).

And so we ask: how does such diversity in users affect LTS evolution, both in early stages (invention, growth) and in later phases (contestation, decline)? Are more elaborate typologies of users needed? What influences the impression held by system builders of who the legitimate users of the system are, including when there are user-system builders?

## Conclusion

**Figure 2.**
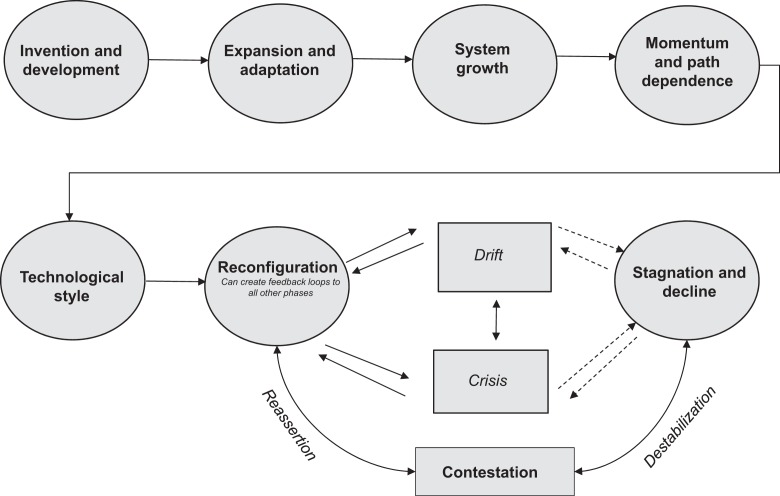
Eight conceptual phases of large technical systems. Source: Authors.

We maintain that many LTS can progress through not only the Hughesian phases of (1) invention and development, (2) expansion and adaptation, (3) system growth, (4) momentum and path dependence, and (5) technological style but also (6) reconfiguration, (7) contestation, and (8) stagnation and decline summarized by Figure 2. Perhaps obviously, not all LTS will ever make it through all eight phases; for example, some can remain in a perpetual cycle between contestation and reconfiguration. Moreover, the phases are not always sequential; feedback loops often exist between phases that push systems across the spectrum, not necessarily in a linear order, something depicted in the figure with its arrows. Nor are the phases as neat as we imply; we deploy them here to illustrate where a particular set of mechanisms are most dominant; elements of phases or particular mechanisms are often present across other phases and mechanisms. The dotted areas between contestation and stagnation and decline also indicate that many systems will never enter full decline—they will simply be shocked back into reconfiguration.

The cases discussed in this study illustrate not only the presence of these eight phases but also the complexity of paths of progression and change. Our framework builds on these complexities, emphasizing the dynamism and coevolution of our phases of development, which is why the dotted lines in the Figure 2 indicate that only some LTS progress to actual decline. Nevertheless, there is a stylized element to our eight phases—not all LTS may traverse through them, and development will often be sporadic and episodic. Put another way, both the ascent and the fall of LTS will shift based on variation and selection processes. Also, evolutionary change includes variation, selection, and retention.

Furthermore, our study suggests that path dependence should not be understood to mean that systems are simply locked in or obdurate, but that continuity is a constant mobilization of resources by those that are advantaged by the present system, who may seek to protect and maintain their advantages (Sorensen 2015, 28). Moments of reconfiguration, contestation, and decline can “open them up” to inquiry, challenge, and significant change. Van der Vleuten (2006) adds that as mature systems may resist change, strategies do exist to exert pressure and alter system dynamics—these can range from setting up protected spaces for niches to grow, facilitating participative technology assessment methods, and identifying internal points of pressure such as congestion, or external points of pressure such as major political events or crises.

Future research is needed to test, validate, and further explore insights offered by our framework. More refined typologies of LTS subcomponents, layers, functions, and patterns of development deserve consideration. More reflexive thinking on the temporalities of LTS evolution as well as the complexity of users would also be fruitful. An important next step would be tracing the progression of a single system, or a series of LTS, through our eight phases. Another would be showing the coevolution of systems, and how different LTS may evolve together in interdependent or independent ways. At times, they may overlap at junctions. Yet another would be testing our framework with original data gleaned from interviews, surveys, or focus groups—input that could come from system builders or historians. Further paths for future development could include developing the framework into an active tool to assist policy intervention in these notoriously complicated and vitally important systems. These ideas underscore the themes of nonlinearity, variation, and complexity in LTS evolution.

The previous century has shown that fully established LTS rarely undergo full decline or displacement. As a result, understanding the mechanisms and characteristics of reconfiguration and contestation, introduced in this paper, is crucial to supporting and interacting with these society-shaping systems.
